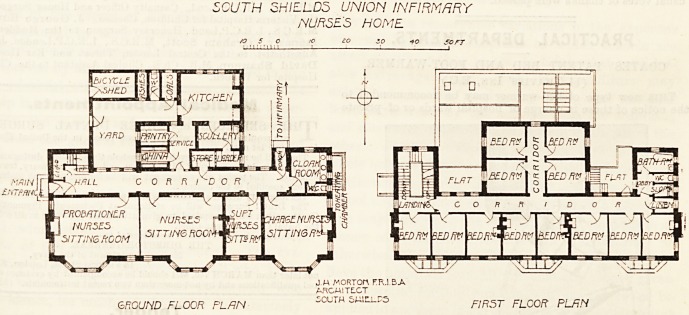# Nurses' Home at the South Shields Union Infirmary

**Published:** 1905-02-18

**Authors:** 


### NURSES' HOME AT THE SOUTH SHIELDS UNION INFIRMARY.

HITHERTO the nurses in this infirmary have had their quarters in the wards, but the guardians have lately shown their interest in the nursing staff, and their consideration has borne fruit in the shape of a good home for the nurses, placed about 160 feet from the main institution and to the south thereof. Altogether the new building gives accommodation for twenty-four nurses and domestics, and the accommodation provided is really good.

The ground floor has at its west side the main entrance, and from this point a corridor runs east for the entire length of the home. At its west end there is a projection northwards containing the staircase, and a similar projection at its east end provides for the cloak-room and closets. Near the cloak-room is a porch leading to the covered way to the infirmary, and the cloak-room is of large size, and is intended as a sort of dressing-room in which the nurses can divest themselves of their hospital clothing before entering their sitting-rooms. This is an admirable arrangement. To the south of the main corridor are the nurses' sitting-room and dining-room. These rooms have been wisely provided with a collapsible partition, so that if need be both rooms can be thrown into one and used as a lecture hall or amusement room. Next the dining-room is the lady superintendent's sitting-room, and further east is the charge-nurses' sitting-room.

North of the main corridor is the well-arranged kitchen department. A bicycle-room occupies one corner of the kitchen-yard, but it is properly reached from the outside of the yard.

The first floor has twelve bedrooms and an excellent bathroom, closet, sinks, and a linen-room.

Access to the second floor, which contains similar accommodation to the first floor, is obtained by a fire-proof staircase, and in addition to this both of the upper floors are provided with fire escapes.

The home is lighted throughout with electricity. The electric mains, as well as the steam and hot-water pipes, are placed under the main corridor, and are easily reached in the event of repairs or renewals. All the sitting-rooms and many of the bedrooms have open fire-places, and, as a heating-chamber is shown it is to be presumed that hot water or steam coils are used in the corridors and wherever else they may be necessary.

The south elevation, although plain, as indeed such union infirmary buildings ought to be, is quite pleasing.

The architect was Mr. J. H. Morton, of South Shields, the contractor Mr. John Moore, and the clerk of the works Mr. J. T. Robson. The cost was £4,200. This sum cannot be looked upon as at all extravagant, as there is no evidence of curtailing the accommodation in any way, and it would work out at £175 a bed, but this is not quite a fair way of estimating the cost of such a building because the administration department and the warming and lighting plant have to be almost as large for twenty-four beds as for double the number.

## Figures and Tables

**Figure f1:**